# Blood sampling for research purposes in children: a practical guide to volume limits and minimisation strategies

**DOI:** 10.1007/s00431-026-07583-1

**Published:** 2026-10-29

**Authors:** Janez Jazbec, Barbara Faganel Kotnik, Tomaž Prelog, Marko Pokorn

**Affiliations:** 1 Department of Paediatric Haematology and Oncology, University Medical Centre Ljubljana, University Children’s Hospital, Ljubljana, Slovenia; 2 Faculty of Medicine, Department of Paediatrics, University of Ljubljana, Ljubljana, Slovenia; 3 University Medical Centre Ljubljana, University Children’s Hospital, Ljubljana, Slovenia

**Keywords:** Blood sampling, Blood volume, Children, Research ethics, Microsampling, Pharmacokinetics

## Abstract

Blood sampling performed only for research purposes usually offers no direct benefit to the child, and children’s limited total blood volume and susceptibility to anaemia call for a cautious, standardised approach. This review summarises published recommendations on blood sampling volumes for research purposes in children and translates them into practical guidance for protocol design and everyday research practice. Guidance from WHO, the European Commission, PedCRIN and ICH documents was synthesised together with current empirical literature on the safety of blood sampling and on volume-minimisation techniques in paediatric studies. Published recommendations are heterogeneous and rest on limited evidence. European recommendations propose that research-related blood loss should as a general rule not exceed 1% of total blood volume (TBV) at any single time point and 3% of TBV over 4 weeks. We present these as reference values in a weight-based table, applied over a rolling 4-week window. In children with clinically significant anaemia, research-only sampling should be deferred or individually justified. Residual diagnostic samples, small-volume collection tubes, sparse sampling with population pharmacokinetic modelling, and volumetric microsampling (VAMS/DBS) substantially reduce blood burden, although microsampling has analytical limitations. Practical tracking of cumulative blood volume and coordination between teams are described. Parental informed consent must be complemented by the child’s assent, sought according to age and maturity and checked during the study.

*Conclusion*: These reference values and minimisation strategies offer a practical starting point for protocol design and ethics review. They are not proof of safety and need individual adjustment in sick children.

**Table:** 

**What is Known:** • *Published recommendations on research blood sampling in children are heterogeneous; European recommendations propose ≤ 1% of total blood volume at a single time point and ≤ 3% over 4 weeks.* • *Evidence supporting these thresholds is limited.*
**What is New:** • *This review translates these recommendations into a practical framework: a weight-based reference table, rolling 4-week tracking of research blood loss alongside clinical sampling, and coordination between clinical and research teams.* • *It summarises residual-sample and microsampling strategies, including their limitations.*

**What is Known:**

• *Published recommendations on research blood sampling in children are heterogeneous; European recommendations propose ≤ 1% of total blood volume at a single time point and ≤ 3% over 4 weeks.*

• *Evidence supporting these thresholds is limited.*

**What is New:**

• *This review translates these recommendations into a practical framework: a weight-based reference table, rolling 4-week tracking of research blood loss alongside clinical sampling, and coordination between clinical and research teams.*

• *It summarises residual-sample and microsampling strategies, including their limitations.*

## Introduction

Blood sampling for research purposes is an important component of clinical and translational studies. In children, sampling done only for research purposes usually brings no direct benefit, although some research samples, such as drug concentrations used for dose adjustment, may also guide treatment. Due to a limited total blood volume and an increased susceptibility to anaemia, a particularly cautious and standardised approach is required in the paediatric population [1]. The aim of this review is to provide an overview of published recommendations and to formulate practical guidance on blood sampling volumes for research purposes in children. The guidance presented here draws on international guidance documents (WHO, European Commission, PedCRIN, ICH), current empirical literature, and the authors’ clinical and research experience in paediatric haematology and oncology.

## General principles

The choice of sampling method and site depends on the volume of blood needed and on the child’s age and weight [2]. When designing a research protocol, the volume of blood collected must be limited to the minimum amount necessary to achieve the scientific objectives, with due regard to the child’s health status [3, 4]. Cumulative burden is the total volume of blood drawn from a child over a defined period, including clinical and research samples and any discarded blood. The protocol should state the maximum volume per draw, per 4 weeks and for the whole study, and the minimum interval between draws.

Contemporary European initiatives in paediatric clinical research, including the PedCRIN initiative, further emphasise the principle of biological sample minimisation and the need for explicit justification of each blood draw in the research protocol [3].

Any research involving blood sampling in children must be reviewed by the competent Ethics Committee, which, on the basis of risk assessment and the scientific value of the study, may require additional restrictions or modifications [4].

## Blood sampling volumes in children

Most recommendations are based on a percentage of total blood volume (TBV), which is about 80–90 mL/kg in children and about 100 mL/kg in neonates [4]. Published recommendations are heterogeneous. Policies reviewed by Howie ranged from 1–5% of TBV within 24 h to up to 10% of TBV over 8 weeks, and the evidence behind them is limited [1]. The 2017 European recommendations propose that research-related blood loss, including waste, should as a general rule not exceed 1% of TBV at any single time point and 3% of TBV over 4 weeks. They state that these values are not evidence-based and may be excessive in infants who already need much clinical sampling [4]. These thresholds should therefore be regarded as reference values rather than universal regulatory limits.

Observational data from three clinical trials suggest that repeated venous draws of up to about 3% of TBV, taken at three-monthly intervals, may be tolerated in selected children aged 6 months to 12 years without signs of anaemia or hypovolaemia (141 children, 629 draws) [5]. These data cannot be extrapolated to children with active anaemia, critical illness or conditions associated with impaired haematopoietic recovery.

These limits are general rules. An ethics committee may approve higher research volumes when they are scientifically justified and the risk is minimised, for example in paediatric oncology, when research samples are taken during clinically indicated procedures and the results may guide the child’s treatment. The justification and the monitoring plan should be stated in the protocol. Blood transfusions or iron supplementation should not be used to justify larger or more frequent research draws [4].

## Timing and cumulative volume

In addition to absolute volume, the interval between individual draws plays a key role. Repeated draws within days, for example daily sampling during an acute illness, add up quickly; in intensive care, children under 10 kg lose the most blood per kilogram of body weight [6]. Research protocols should therefore clearly specify the maximum volume per draw, per 4 weeks and for the whole study. The 4-week limit applies to any rolling 4-week period, not to fixed blocks counted from the start of the study. Before each draw, the research volumes drawn in the preceding 4 weeks are added up and compared with the limit. In longer studies, the same check is repeated before every draw until the study ends. Table 1 gives the resulting reference volumes by body weight.

**Table 1:** Reference blood sampling volumes for research purposes in children by body weight: single time point and any 4-week period

Body weight (kg)	TBV (mL)*	Single time point, ≤ 1% TBV (mL)	Any 4 weeks, ≤ 3% TBV (mL)†
**Neonates (first 28 days): TBV 100 mL/kg**			
2.5	250	2.5	7.5
3	300	3.0	9.0
3.5	350	3.5	10
**Infants, children and adolescents: TBV 80 mL/kg**			
4	320	3.2	9.6
6	480	4.8	14
7.5	600	6.0	18
10	800	8.0	24
12	960	9.6	28
14	1120	11	33
18	1440	14	43
22	1760	17	52
28	2240	22	67
34	2720	27	81
42	3360	33	100
52	4160	41	124
62	4960	49	148

*TBV, total blood volume, calculated according to the European recommendations as 100 mL/kg in neonates and 80 mL/kg in older infants, children and adolescents, the lower end of the 80–90 mL/kg range [4]. In neonates, 1% of TBV therefore equals 1 mL/kg. In neonates who already need much clinical sampling, and in preterm infants, even these volumes may be excessive [4]

†Cumulative research-related blood loss, including waste, in any rolling 4-week period. Volumes below 10 mL are given to one decimal place; larger volumes are rounded down to whole millilitres. All values are reference values, not universal regulatory limits

## Practical tracking of cumulative blood volume

A further practical challenge, distinct from the volume limits themselves, is reliably tracking cumulative blood volume across the rolling 4-week window when clinical and research sampling are performed concurrently by different teams for different purposes. No single service typically has automatic visibility into every draw a child undergoes, and reliance on informal recall or retrospective chart review is prone to error, particularly during longer or more complex admissions.

Institutional experience from analogous clinical settings offers a practical template. Implementation-science-based programmes that combined systematic tracking of blood drawn with structured team coordination have measurably reduced excess phlebotomy and downstream transfusion need in critically ill children, without requiring sophisticated infrastructure [7]. Electronic health record tools can also help: in a neonatal intensive care unit, revised order sets and documentation of the blood drawn reduced laboratory testing and blood loss in preterm infants [8]. Where analytics tools are available, ordered and drawn volumes can be summed automatically into a running total per patient.

At minimum, research protocols should specify who is responsible for maintaining a cumulative log—typically the study coordinator or principal investigator—and require that the running total, converted to %TBV using the child’s current weight, be checked before each new draw against the 1% single time point and 3% four-week limits. Clinical draws in the same period should be recorded in the same log, so that a research draw can be postponed when clinical sampling is heavy. A visible flag in the patient’s chart or research file, accessible to any team drawing blood, is a practical low-technology alternative where electronic tracking is unavailable.

## Special circumstances

In children with pre-existing anaemia, chronic diseases, or coagulation disorders, an individualised approach is required and the permissible sampling volume may be substantially lower than the generally recommended limits. The proposed volume should be explicitly justified in the research protocol, with appropriate clinical monitoring ensured [3, 4]. In children with clinically significant anaemia, research-only blood sampling should generally be deferred or individually justified by the investigator together with the treating team. A haemoglobin concentration below 80 g/L may be used as a pragmatic institutional review threshold, rather than as a universal contraindication. Lower limits are advisable in sick children, particularly those with low haemoglobin or reduced blood volume [1].

## Use of residual diagnostic samples (left-over sampling)

The ethically and practically most important strategy for reducing the burden on the child is the use of residual samples obtained for diagnostic purposes (left-over or residual sampling). Where scientifically and logistically feasible, the research protocol should prioritise the use of samples that would otherwise be discarded after completion of clinical analyses [3]. This approach does not increase the total sampling volume and is consistent with the principle of sample minimisation explicitly recommended by the PedCRIN initiative [3]. The European recommendations also advise using blood remnants whenever possible [4].

The use of residual samples must be described in the protocol and in the informed consent form. Separate consent is needed only when samples are to be stored for future research not described in the protocol, or when national law requires it. This approach is of particular importance in preterm infants and low birth weight neonates, in whom iatrogenic blood loss from repetitive laboratory testing is a major contributor to anaemia of prematurity and the need for red blood cell transfusions [8].

## Coordination between clinical and research teams

The availability of residual diagnostic material cannot be assumed. When the clinical team has already drawn the volume clinically needed, little or no residual material may remain. In intensive care, clinical sampling alone can be large enough to contribute to anaemia and transfusion need [6, 7].

Two principles should guide institutional practice. First, the research limits in Table 1 apply to research-related blood loss, but the child’s total burden also includes clinical sampling. A study that complies with these limits may still add to an already heavy clinical sampling burden. Investigators and ethics committees should therefore assess and monitor research and clinical sampling together [1, 4]. Second, research protocols should specify in advance how sampling will be prioritised when clinical and research sampling compete for the same child.

Practical mechanisms include: deferring the research draw whenever clinical sampling in the same period is heavy; embedding research aliquots within an already clinically indicated venipuncture whenever timing allows, so that a single puncture serves both purposes; and establishing a direct communication channel between teams—such as a shared electronic flag or a designated research liaison—so that clinical staff are aware a child is enrolled in a blood-sampling research protocol. If coordination is not possible, a research-only draw should be postponed rather than add to an already heavy clinical sampling burden. This does not apply to samples needed for the child’s safety or for dose adjustment of the study drug; these are treated as clinically necessary.

## Technical volume-minimisation strategies

### Small-volume collection tubes

The simplest and most immediately applicable volume-minimisation strategy is the use of paediatric small-volume collection tubes. Standard adult tubes (typically 3.5–10 mL) often lead to drawing more blood than the analysis requires; in one paediatric intensive care unit, the excess drawn was on average about twice the volume requested by the laboratory [6]. Paediatric tubes (0.5–1 mL) reduce this excess. Tube selection should be reviewed at the protocol design stage and minimum required volumes should be specified for each assay, including common panels (full blood count, biochemistry, coagulation).

### Sparse sampling and population pharmacokinetics

Sparse sampling is an approach in which only 2–3 blood samples are collected per individual child instead of the conventional 10–20. The overall pharmacokinetic (PK) profile is then reconstructed at the group level using a population PK (PopPK) model, in which each child contributes samples at different time points; combined with nonlinear mixed-effects modelling, this framework enables reliable estimation of PK parameters from a substantially reduced total sampling burden across the cohort [9]. The approach is now an established standard in paediatric oncology and is explicitly recommended by the ICH E11(R1) guideline. The optimal schedule of time points is determined using model-informed (optimal) design, which applies mathematical optimisation to maximise information content at minimum sampling volume.

### Volumetric absorptive microsampling (VAMS) and dried blood spots (DBS)

Volumetric microsampling is a technique in which whole blood is collected by fingerprick or heelprick directly onto an absorbent tip (e.g. Mitra® VAMS device) or filter paper card (dried blood spot, DBS), with a total volume of only 10–30 µL per time point. This volume is one to three orders of magnitude lower than a conventional venous draw and eliminates the need for venipuncture in many PK applications. The technique was validated and deployed across two global phase III paediatric trials of baricitinib in children aged 2–18 years, where each time point required only 2 × 10 µL of blood, demonstrating feasibility in regulatory-grade studies [10]. ICH M10 (bioanalytical method validation) sets out validation requirements for dried matrix methods, and ICH S11 (nonclinical safety studies in juvenile animals) encourages microsampling. VAMS and DBS have been validated for a range of analytes including small molecules, biologics, and immunosuppressants. When combined with sparse sampling, microsampling approaches greatly reduce the cumulative blood burden in pharmacokinetic studies.

Microsampling has limitations. In the baricitinib trials about 7% of microsamples could not be analysed, mostly because of incomplete sampling, despite site training [10]. Each assay must be validated for the microsample matrix. With dried blood spots, the haematocrit can bias the measured concentration, and analytes present at low concentrations may fall below the sensitivity of the assay.

### Capillary microsampling

Capillary microsampling via heelprick (from birth to about 6 months of age) or fingerprick (in older children) [2] allows collection of 50–200 µL of whole blood without venipuncture. This approach is particularly useful in neonates and infants, where venous access carries procedural risk and repeat punctures are poorly tolerated. Capillary samples are compatible with many routine and research assays and can be collected by nursing staff as part of routine care, further reducing the procedural burden. Research protocols should specify whether capillary sampling is acceptable for the intended assay and confirm analytical validation for the matrix. Heel and finger pricks are painful. Pain should be prevented and treated during all research blood draws, for example with oral sucrose in newborn infants and with topical anaesthesia before venipuncture in older children [2, 4, 11].

All volume-minimisation strategies should be evaluated during protocol design and their rationale communicated to the ethics committee in the protocol submission. Whoever designs the protocol, whether an industry sponsor, an academic investigator or a consortium, should involve people with paediatric clinical, laboratory and pharmacometric expertise, so that the number and volume of draws are kept to a minimum. Combining approaches—for example, sparse sampling with capillary microsampling using DBS—can reduce the total blood burden of a PK study to a level close to that of background clinical sampling [9, 10].

## Informed consent and child assent

Research blood sampling in children requires informed consent from parents or legal guardians and the child’s assent, in line with national law. Assent should be sought according to the child’s age, maturity and clinical situation rather than a fixed age threshold [4]. It is an ongoing process: the child’s willingness should be checked during the study and, where appropriate, before invasive procedures such as blood sampling, and a child’s dissent should be respected [4].

## Role of ethics committees

Binding European and international regulations do not set uniform numerical limits for the maximum blood sampling volume in children; recommendations emphasise the responsibility of investigators and ethics committees to carry out an individual risk assessment [4]. Ethics committees play a key role in ensuring that the burden imposed on the child is proportionate to the scientific value of the study and that all available measures to minimise risk have been applied. Protocol submissions should explicitly document the volume-minimisation approach adopted and justify any blood sampling not covered by residual or combined clinical draws.

## Conclusion

Recommendations on research blood sampling in children are heterogeneous and rest on limited evidence. We propose a practical framework that uses the European reference values of 1% of TBV at a single time point and 3% over 4 weeks, tracks research blood loss over a rolling 4-week window alongside clinical sampling, and gives preference to residual samples and microsampling. These values are a starting point for protocol design and ethics review, not proof of safety, and they need individual adjustment in sick children.

When pharmacokinetic sampling is unavoidable, the combination of sparse sampling and volumetric microsampling (VAMS/DBS) can greatly reduce blood burden while maintaining analytical quality, provided the assay is validated for the sample matrix [9, 10]. Small-volume collection tubes and capillary microsampling offer further practical reductions applicable across a broad range of study types.

Ensuring informed parental consent and the child’s ongoing, age-appropriate assent is equally essential. Consistent application of this guidance and modern technical strategies supports the ethical and scientifically rigorous conduct of paediatric research and strengthens the trust of children and their legal representatives in research activities.

## Data Availability

No datasets were generated or analysed during the current study.

## Abbreviations

DBS: Dried blood spot
ICH: International Council for Harmonisation
PK: Pharmacokinetic(s)
PopPK: Population pharmacokinetics
TBV: Total blood volume
VAMS: Volumetric absorptive microsampling
WHO: World Health Organization

## Declarations

### Use of generative AI

Generative artificial intelligence tools were used for language assistance and literature search support during the preparation of this manuscript. All scientific content, conclusions, and the final text were reviewed, verified, and approved by the authors. No AI tool is listed as an author.
